# “More” Artificial mRNAs: Beyond the Art of Nature

**DOI:** 10.1002/advs.202521523

**Published:** 2026-02-26

**Authors:** Yuanzhe Cui, Minami Fukui, Hirohisa Ohno, Hirohide Saito

**Affiliations:** ^1^ Center for iPS Cell Research and Application Kyoto University Kyoto Japan; ^2^ Graduate School of Medicine Kyoto University Kyoto Japan; ^3^ Faculty of Medicine Kyoto University Kyoto Japan; ^4^ Institute for Quantitative Biosciences The University of Tokyo Tokyo Japan; ^5^ Department of Bioengineering School of Engineering The University of Tokyo Tokyo Japan

**Keywords:** branched RNA, circular RNA, microRNA‐responsive switch, RNA engineering, RNA therapeutics, self‐amplifying RNA, synthetic mRNA

## Abstract

Messenger RNA (mRNA) has emerged as a versatile platform for gene expression and therapeutic innovation. Early engineering efforts focused on optimizing canonical mRNA components—the 5′ cap, untranslated regions, coding sequence, and poly(A) tail—to enhance stability, translational efficiency, and safety. These refinements culminated in the success of mRNA vaccines and consequently enabled diverse biomedical applications ranging from gene and cell therapy to genome editing. More recently, research has expanded beyond the structural constraints of natural mRNAs, giving rise to non‐canonical architectures, such as circular, branched, self‐amplifying, and lantern‐shaped RNAs. These designs confer novel properties, including resistance to degradation, autonomous replication, and programmable control of translation. Progress in chemical modification, ribozyme engineering, and RNA nanotechnology has further accelerated the diversification of synthetic mRNA. Together with advances in synthesis, purification, and delivery technologies, these innovations are transforming mRNA from a transient messenger into a designable molecular system. This review revisits the evolution of mRNA engineering—from natural optimization to creative structural redesign—and outlines emerging concepts that illustrate how synthetic mRNA is expanding the possibilities of gene expression control.

## Graphical abstract

Inspired by nature yet transcending it, synthetic mRNA is being redesigned beyond the canonical architecture. This review highlights emerging forms—circular, branched, and self‐amplifying mRNAs—that expand stability, persistence, and functional control, illustrating how artificial mRNA is evolving into a new medium for programmable biological expression.

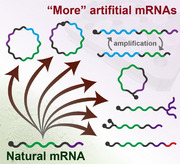

## 1 Introduction

RNAs are broadly categorized as either messenger RNAs (mRNAs), encoding amino acid sequences for protein synthesis, or non‐coding RNAs (ncRNAs), lacking protein‐coding potential. RNA‐based therapeutics have been predominantly focused on non‐coding RNAs, including small interfering RNAs(siRNAs) for silencing target gene expression, ribozymes for cleaving target RNAs, and RNA aptamers for binding to specific target molecules and subsequent functional inhibition [1]. However, this paradigm dramatically shifted with the advent of mRNA vaccines.

Using mRNA as a gene‐expression vector is not a novel concept. Its practical implementation, however, had long been constrained by low stability, high immunogenicity, and technical limitations in the synthesis and delivery of RNA. The discovery that base modification attenuates innate immune responses [2, 3], together with advances in enabling technologies, progressively laid the groundwork for the clinical application of synthetic mRNA. Against this backdrop, the COVID‐19 pandemic catalyzed the rapid development and global validation of mRNA vaccines, elevating synthetic mRNA to a central position in RNA‐based therapeutics.

As a gene expression vector, mRNA offers several advantages [4, 5]. Functioning in the cytoplasm, mRNA does not require nuclear import and is therefore applicable to many cell types, including non‐dividing cells. mRNA sequence design is straightforward, while in vitro transcription (IVT) driven by RNA polymerase is technically simple. In principle, exogenous mRNA poses no risk of integration into the host genome. This genomic safety is crucial, particularly in human therapeutics, and has enabled applications such as vascular endothelial growth factor (VEGF) mRNA therapy for heart failure [6] and reprogramming of human somatic cells into induced pluripotent stem cells (iPSCs) using mRNA encoding Yamanaka factors [7, 8]. Research on synthetic mRNA now extends far beyond vaccines, spanning both basic discovery and clinical development [9].

## 2 In the Image of Nature: Canonical mRNA Engineering

**FIGURE 1:**
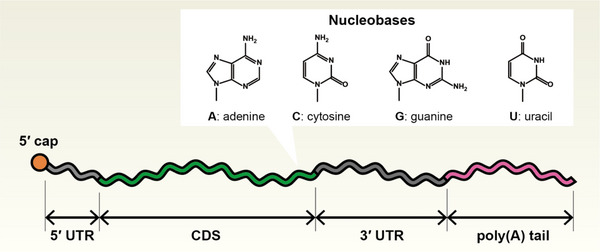
Schematic representation of a canonical eukaryotic mRNA. The molecule consists of a 5′ cap structure, a 5′ untranslated region (UTR), a coding sequence (CDS), a 3′ UTR, and a poly(A) tail. Representative nucleobases (A, C, G, U) are shown.

Synthetic mRNAs hold great promise for a wide range of applications. However, issues such as limited chemical and biological stability and the consequent short‐term action still require improvement. In some applications, protein expression levels are too low to exert therapeutic effects. Furthermore, for clinical or in vivo use, minimizing adverse reactions poses a critical challenge. Although diverse strategies have been explored to address these issues, this review focuses specifically on the engineering of the RNA molecule itself, presenting several key structural components (Figure 1).

### 2.1 Chemical Modifications of Nucleobases

Exogenous RNA strongly stimulates the immune system. While this adjuvant activity can be beneficial in some contexts, excessive immune responses often lead to undesired effects, including cell death. A variety of elements, including Toll‐like receptors, RIG‐I, and PKR, are involved in immune responses to foreign RNA [10]. Karikó and colleagues discovered that replacing standard nucleotides in mRNA with modified bases, such as 5‐methylcytosine (m^5^C) and pseudouridine (ψ), effectively suppresses these immune responses [2, 3]. These modifications resulted in reduced cytotoxicity and improved protein expression. Subsequently, replacing uridine with *N* ^1^‐methylpseudouridine (m^1^ψ) was found to be more effective than replacing it with ψ [11], and is now widely used in mRNA vaccines and other applications.

Research is ongoing to identify additional beneficial base modifications. The efficacy of *N* ^4^‐acetylcytosine, which is naturally present in human cells [12], 5‐fluorocytosine [13], and 2‐aminoadenine, a base found in phages infecting cyanobacteria [14], has been reported. Nevertheless, m^1^ψ has demonstrated high efficacy regardless of mRNA sequence or cell type, and can be effective on its own, making it the current standard in synthetic mRNA design.

Although most efforts have focused on nucleobase modifications, recent studies have demonstrated that chemical modification of the ribose moiety—particularly at the 2′ position—can also modulate RNA stability, immunogenicity, and cellular uptake. For example, “RNA cloaking” strategies that introduce reversible 2′‐OH polyacylation to transiently shield RNA from nuclease degradation and immune recognition have been reported [15]. In addition, systematic incorporation of 2′‐fluoro and 2′‐O‐methyl nucleotides has been shown to enhance stability in serum and translational performance while attenuating innate immune responses [16]. These ribose‐based chemistries expand the design space of synthetic mRNA beyond nucleobase modification alone.

### 2.2 5′ Cap

Eukaryotic mRNAs, including those of humans, possess a *N* ^7^‐methylguanosine (m^7^G) cap structure at their 5′ ends. This cap interacts directly with the translation initiation factor eIF4E, thus playing a central role in initiating translation. In addition, it prevents 5′→3′ exonucleases from degrading the mRNA. One of the initial challenges in the field of synthetic mRNA was the efficient generation of capped RNAs. This was initially addressed by adding a dinucleotide cap analog—consisting of m^7^G linked to G via a 5′–5′ triphosphate bridge—into the in vitro transcription reaction, allowing it to be competitively incorporated at the 5′ end during transcription initiation. Subsequently, methylation of the ribose's 3′‐hydroxyl group on the m^7^G moiety prevented reverse incorporation of the cap structure [17]. This modification, known as the anti‐reverse cap analog (ARCA), became a widely adopted method for producing capped synthetic mRNA. With this approach, various chemical modifications can be made at the cap site using different chemically synthesized cap analogs. For example, phosphorothioate modification of the triphosphate bridge between the cap and the mRNA or ribose modification of the cap structure can enhance translational activity [18]. Later, a trinucleotide cap analog was developed, which improved yields of capped RNA and reduced immunogenicity through 2′‐O‐methylation of the +1 nucleotide (cap‐1 structure) [18]. For trinucleotide cap analogs, chemical modification can also be introduced at the first transcribed nucleotide (+1 position) of the RNA itself. It has been shown that N6‐methyladenosine at the +1 position confers greater resistance to Dcp2‐mediated decapping and stabilizes mRNA compared with unmodified adenosine [19]. On this basis, trinucleotide cap analogs incorporating m6A at the +1 position were developed and were found to exhibit improved stability against decapping enzymes [20]. These advances illustrate how fine chemical tuning of the cap structure can balance translational efficiency, stability, and immune compatibility—key parameters for modern mRNA therapeutics.

Beyond improving stability and translational efficiency of synthetic mRNA, numerous applications of cap modifications have been reported, such as fluorescent dyes [21, 22], the development of a photoresponsive translation control system by adding a specific light‐sensing functional group that inhibits interaction with eIF4E [23, 24], or modifying the cap with hydrophobic tags removable by light irradiation to produce high‐purity mRNA [25].

Cap structures can also be enzymatically introduced post‐transcriptionally. Vaccinia virus capping enzyme (VCE), which is commercially available and exhibits high capping efficiency, is widely used for this method [26]. It is also possible to incorporate modified nucleotides at the cap site by using VCE, enabling the synthesis of non‐natural caps with high translational activity or fluorescently labeled caps [27]. More recently, capping enzymes derived from Faustovirus, which possess high activity across a wider temperature range than VCE [28], have become commercially available, further expanding options for synthesizing capped RNA.

### 2.3 UTRs: 5′ and 3′‐ Untranslated Regions

The 5′ and 3′ untranslated regions (UTRs), located upstream and downstream of the coding sequence (CDS), respectively, regulate mRNA stability, translational efficiency, and subcellular localization [29, 30, 31]. UTRs derived from α‐ or β‐globin mRNA, known to have long half‐lives in human cells, are widely used in synthetic mRNAs to enhance stability. Systematic screening of native mRNAs has also yielded UTRs with superior performance, some of which were incorporated into COVID‐19 vaccines [32]. More recently, machine‐learning approaches have been used to explore the vast landscape of UTR sequences and to design sequences that maximize stability and translational efficiency [33]. The rapid growth of ML/AI suggests that AI‐assisted mRNA design may become standard practice in the future. Beyond sequence optimization, “additive” engineering has been reported to enhance translational efficiency by introducing translation initiation factor eIF4G‐binding sequence [34], whereas a “subtractive” strategy regards UTRs as dispensable sources of uncertainty and minimizes them to 4 nucleotides [35]. Together, these approaches underscore that UTRs are not passive flanks but programmable control elements.

**FIGURE 2:**
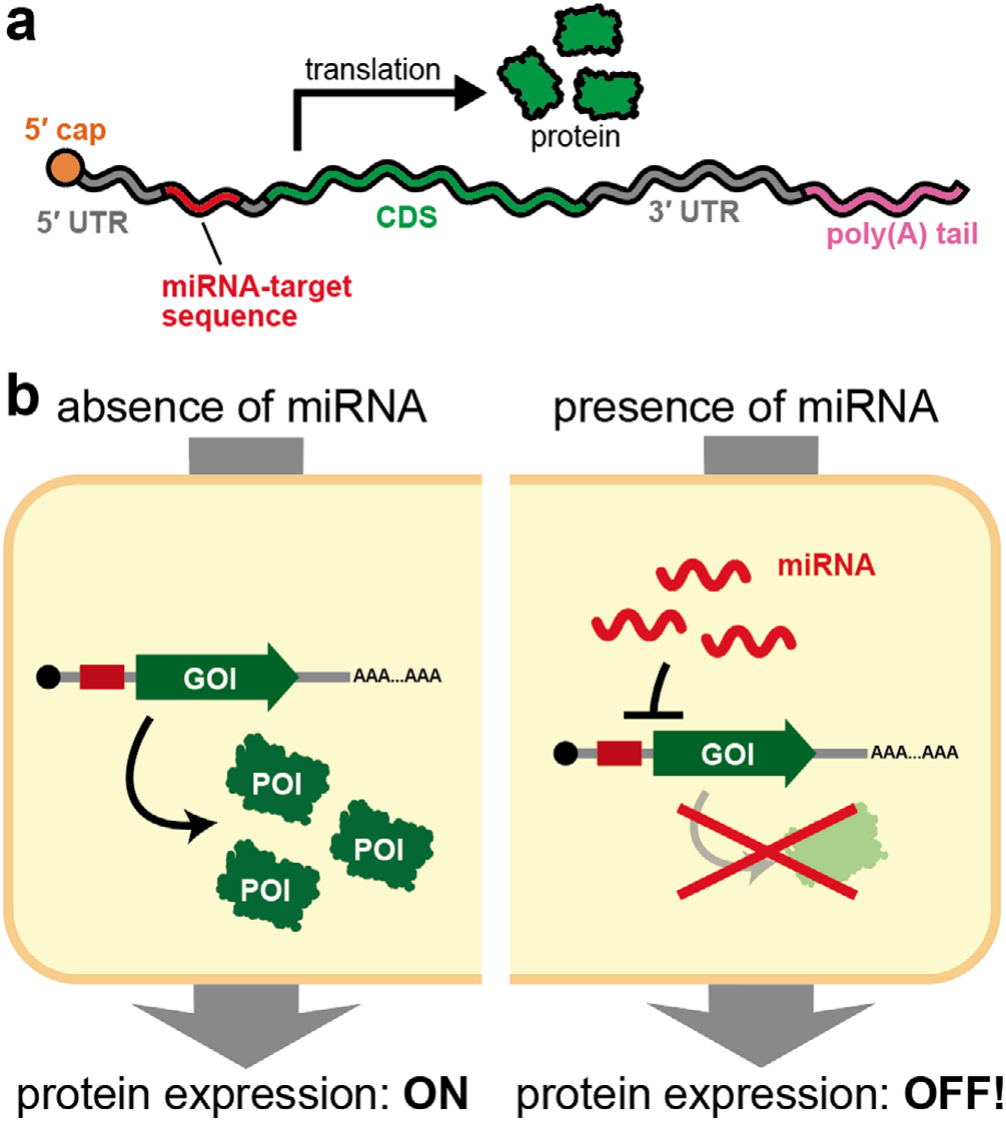
MicroRNA‐responsive OFF switch. (a) The microRNA‐responsive OFF switch is a synthetic mRNA that contains a sequence complementary to an arbitrary microRNA in its untranslated region (UTR). (b) In the absence of the target microRNA, translation proceeds as in conventional synthetic mRNAs, leading to expression of the encoded protein (ON state, left). In the presence of the cognate microRNA, cleavage or translational repression of the switch RNA occurs, resulting in suppression of protein expression (OFF state, right).

UTRs can also be engineered with functional RNA sequences to modulate protein expression. A variety of systems have been reported to regulate translation initiation or stability of synthetic mRNA in response to proteins or small molecules [36, 37]. Here, we highlight a microRNA‐responsive OFF switch developed by our group, for its generality and ease of design. MicroRNAs (miRNAs) are short non‐coding RNAs of ∼22 nucleotides in length in eukaryotes [38]. Transcribed from genomic loci, primary transcripts undergo multistep processing to yield mature miRNAs. They are consequently loaded into the RNA‐induced silencing complex (RISC), which, via base pairing with complementary sites in target mRNAs, represses translation or promotes mRNA decay, thereby down‐regulating gene expression. Leveraging this mechanism, Endo et al. introduced a complementary target site for an arbitrary miRNA into the UTR, yielding an ON state in the absence of the cognate miRNA and an OFF state when it is present (Figure 2), hereafter referred to as the microRNA‐responsive OFF switch [39]. More than 2,500 miRNAs have been identified in humans, and their expression profiles vary by cell type, developmental stage, and physiological state [40, 41, 42]; thus, miRNAs can serve as markers for distinguishing cell populations. In practice, the miRNA‐responsive OFF switch restricts expression of exogenous proteins to target cells, enabling cell‐type identification and selection based on endogenous miRNA activity. Similar microRNA‐based detargeting strategies have been widely adopted to reduce off‐target expression in specific tissues, most notably by incorporating miR‐122 target sites to suppress hepatocyte expression, as demonstrated by Jain et al. [43] and related studies. In addition, similar systems have been incorporated into vaccines to mitigate side effects [44]. Accordingly, miRNA‐responsive gene expression control strategies, represented by OFF‐switch systems, enable cell‐type‐specific regulation of gene expression and facilitate selective expression of target mRNAs.

### 2.4 CDS: Coding Sequence Region

In synthetic mRNAs, the coding sequence (CDS) is constrained by the target protein's amino acid sequence and, therefore, sequence engineering is limited to synonymous codon substitutions. However, codon identity has a significant impact on protein expression: favoring optimal codons can enhance translational output by accelerating elongation. Because slower elongation promotes mRNA decay, codon optimization is also associated with increased mRNA stability [45]. Motivated by evidence that intramolecular mRNA structure correlates with stability [46], recent studies have designed coding sequences with explicit consideration of predicted secondary structure and related features [47, 48].

### 2.5 Poly(A) Tail

The poly(A) tail participates in the formation of the translation initiation complex through interactions with poly(A)‐binding proteins. It also regulates mRNA stability, as the shortening of poly(A) tails by deadenylases leads to mRNA degradation [49]. In general, longer poly(A) tails confer greater mRNA stability and higher protein expression levels. However, due to synthetic constraints, poly(A) tails of approximately 100–140 nucleotides are commonly used.

To protect synthetic mRNA from degradation by deadenylases, various modifications of the 3′ end have been reported, including incorporation of non‐natural nucleotides [50], covalent attachment of fluorescent dyes [51], introduction of phosphorothioate modifications within the poly(A) tail [52], and combinations of phosphorothioate modifications in the last few nucleotides with 3′–3′ thymidine linkages [53]. Although these modifications increase the complexity of synthesis, they can effectively improve synthetic mRNA stability.

## 3 Beyond Nature's Blueprint: Non‐Canonical Architectures of Synthetic mRNA

While the previous section focused on engineering strategies that refine and optimize canonical mRNA components—such as the 5′ cap, untranslated regions, coding sequence, and poly(A) tail—recent advances have moved beyond the constraints of natural architecture. Researchers are now exploring artificial mRNAs that adopt novel structural formats, achieve extended stability, or even possess autonomous functions such as self‐replication. These “non‐canonical” mRNAs expand the conceptual boundaries of RNA design, transforming it from mere optimization of natural features into a creative platform for building new molecular architectures. In this section, we highlight emerging examples of such designs, including circular, branched, self‐amplifying, and lantern‐shaped mRNAs, as well as RNA switches that dynamically control translation.

### 3.1 Circular mRNA

**FIGURE 3:**
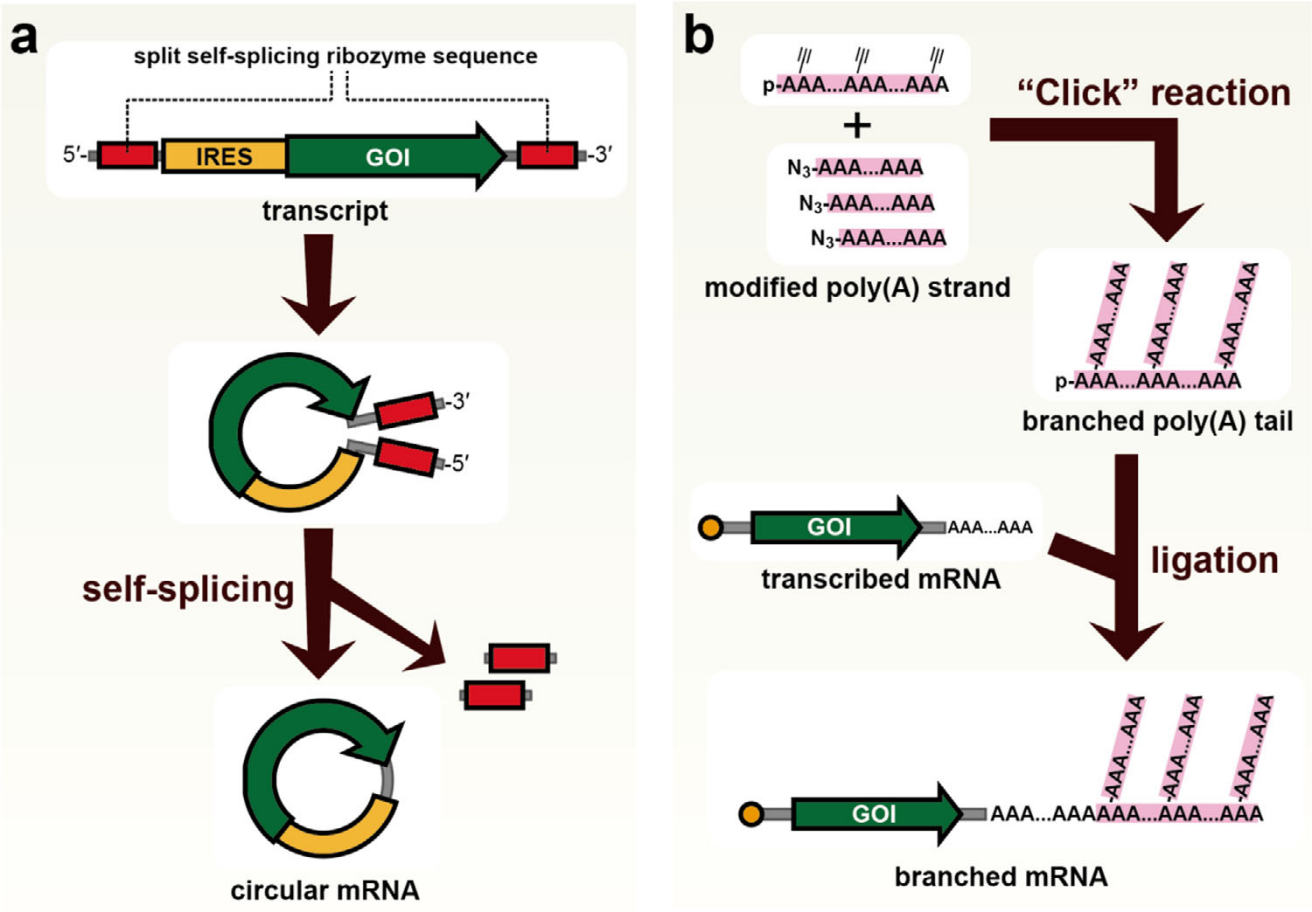
Circular and branched mRNAs. (a) Circular mRNA. The RNA is transcribed to contain split group I intron (self‐splicing ribozyme) sequences, an internal ribosome entry site (IRES) for translation initiation, and a coding region for the desired protein. After transcription, self‐association of the ribozyme sequences at both termini activates the ribozyme, and the self‐splicing reaction removes the ribozyme segments while ligating the adjacent sites, thereby producing circular mRNA. (b) Branched mRNA. Poly(A) chains site‐specifically modified with functional groups for conjugation are chemically synthesized and connected by click chemistry to form a branched poly(A) structure. This branched poly(A) tail is then ligated to the 3′ end of an in vitro transcribed mRNA, yielding a branched mRNA molecule.

Biological instability is the main challenge in the application of artificial mRNA. Unlike vaccines, which only require transient antigen expression, applications such as gene therapies require long‐lasting effects. To address this issue, circular synthetic mRNA, in which RNA ends are covalently joined to resist exonuclease degradation, is drawing attention. The strategy to stabilize RNA by circularization has been adopted for relatively short RNA molecules, such as siRNA [54] and aptamers [55]. In these cases, RNA was circularized by enzymatic ligation using protein‐based enzymes such as ligases. However, the efficient circularization of long RNAs such as mRNAs using similar methods has proven difficult. Wesselhoeft et al. demonstrated the utility of circular mRNA and successfully achieved practical circularization efficiently [56]. They focused on group I introns, a type of ribozyme that performs self‐splicing, by splitting the ribozyme, placing each half at opposite ends of the RNA, and optimizing the surrounding sequences (Figure 3a). The split ribozymes spontaneously assemble in solution and catalyze a splicing reaction, generating circular RNA and ribozyme fragments. Through this mechanism, they efficiently circularized even larger RNAs, containing large genes such as Cas9. Since circular mRNA lacks ends, it does not have a 5′ cap structure. To initiate translation, an internal ribosome entry site (IRES) is inserted to allow cap‐independent translation. These circular mRNAs expressed protein for several days and showed expression for several‐fold longer duration than linear mRNAs. High durability of circular mRNA was also confirmed in vivo [57].

The synthetic method and protein expression level remain challenges for circular mRNA. The longer the RNA, the lower the efficiency of circularization. Additionally, sequences used to promote circularization reactions may remain after ligation and trigger immune responses. Therefore, efforts are being made to design ribozymes and surrounding sequences that can achieve high circularization efficiency without leaving extraneous sequences after the reaction [58, 59]. Furthermore, IRES‐mediated translation is generally less efficient than cap‐dependent translation. Research is ongoing to identify highly active IRES elements or engineer existing IRES to enhance protein expression [60]. Recently, chemical approaches have also been revisited. Wasinska‐Kalwa et al. reported a novel method based on intramolecular reductive amination between a 5′‐ethylenediamine (EDA) modification and a periodate‐oxidized 3′ end [61]. They further demonstrated the construction of cap‐containing circular RNA using an EDA‐modified m^7^G cap.

Since circular mRNAs have enhanced stability, their side effects and off‐target activity could be considerable. To address this issue, we have worked to control protein expression from circular mRNA in a cell‐type‐specific manner [62]. By incorporating sequences complementary to target microRNA into the untranslated regions of circular mRNA, we created a “microRNA‐responsive OFF switch,” in which protein expression is suppressed in the presence of corresponding microRNA. Similar to linear artificial mRNA, this “microRNA‐responsive OFF switch” expressed proteins only in the absence of target microRNA, enabling the construction of circular mRNAs that function only in target cells.

Despite their improved stability, these limitations have important implications for therapeutic translation in vivo. Lower translational efficiency mediated by IRES elements may necessitate higher RNA doses to achieve therapeutic protein levels, which in turn can increase the risk of off‐target expression and innate immune activation. In addition, residual ligation or ribozyme‐derived sequences and altered RNA topology may enhance innate sensing or alter tissue distribution, potentially limiting cell‐type specificity and safety. Together, these factors highlight that, although circular mRNAs are promising for sustained expression, careful optimization of dose, delivery, and immunogenicity will be essential for clinical applications.

### 3.2 Branched RNA

In polymer chemistry, branched architectures such as dendrimers are well known. Similarly, in biological systems, branched RNA structures occur naturally in intron lariats formed during pre‐mRNA splicing. Recently, the synthesis of synthetic mRNAs with branched structures has been reported [63]. Chen et al. chemically synthesized poly(A) chains and connected them by click chemistry and enzymatic ligation to construct branched mRNAs bearing multiple poly(A) tails (Figure 3b). Optimization of the number and chemical modification patterns of poly(A) tails more than doubled the duration of protein expression compared with conventional linear mRNAs. Branched mRNAs exhibited improved in vivo stability and also enabled efficient genome editing in mice with low doses of Cas9 mRNA. Subsequently, they reported additional examples of branched mRNAs, including a dual‐capped (“twin‐headed”) RNA and a circular RNA with a branched cap resembling the letter “Q” [64]. Fukuchi et al. also described the construction of capped circular RNA through enzymatic ligation using a branched cap‐containing RNA fragment [65].

However, the synthetic complexity of branched mRNAs also poses practical challenges for translational development. Multi‐step chemical conjugation and enzymatic ligation can limit scalability and complicate large‐scale manufacturing. In addition, the presence of multiple branch points and chemically modified linkages makes purification, analytical characterization, and regulatory comparability to conventional linear mRNA more challenging. While branched mRNAs offer unique functional advantages, clinical translation will likely require substantial advances in manufacturing and quality control.

Nevertheless, while the complexity of synthesis raises questions about practical applicability, the branched topology represents a novel molecular architecture with vast potential for diversification. It poses fundamental questions such as how far mRNA structure can deviate from its natural form and to what extent such unconventional designs can enhance the functional performance of mRNA.

### 3.3 Self‐Amplifying RNA

Although circular and branched mRNA are more stable than conventional linear mRNA, they are gradually degraded over time. Moreover, as cells divide, the intracellular synthetic mRNA is also distributed between daughter cells, which reduces its concentration by half. Thus, limited persistence represents an unresolved issue, even for circular or branched mRNAs, as their effects diminish over time in different manners. A promising solution to this problem is self‐amplifying RNA (saRNA), also referred to as self‐replicating RNA (srRNA) or replicon.

**FIGURE 4:**
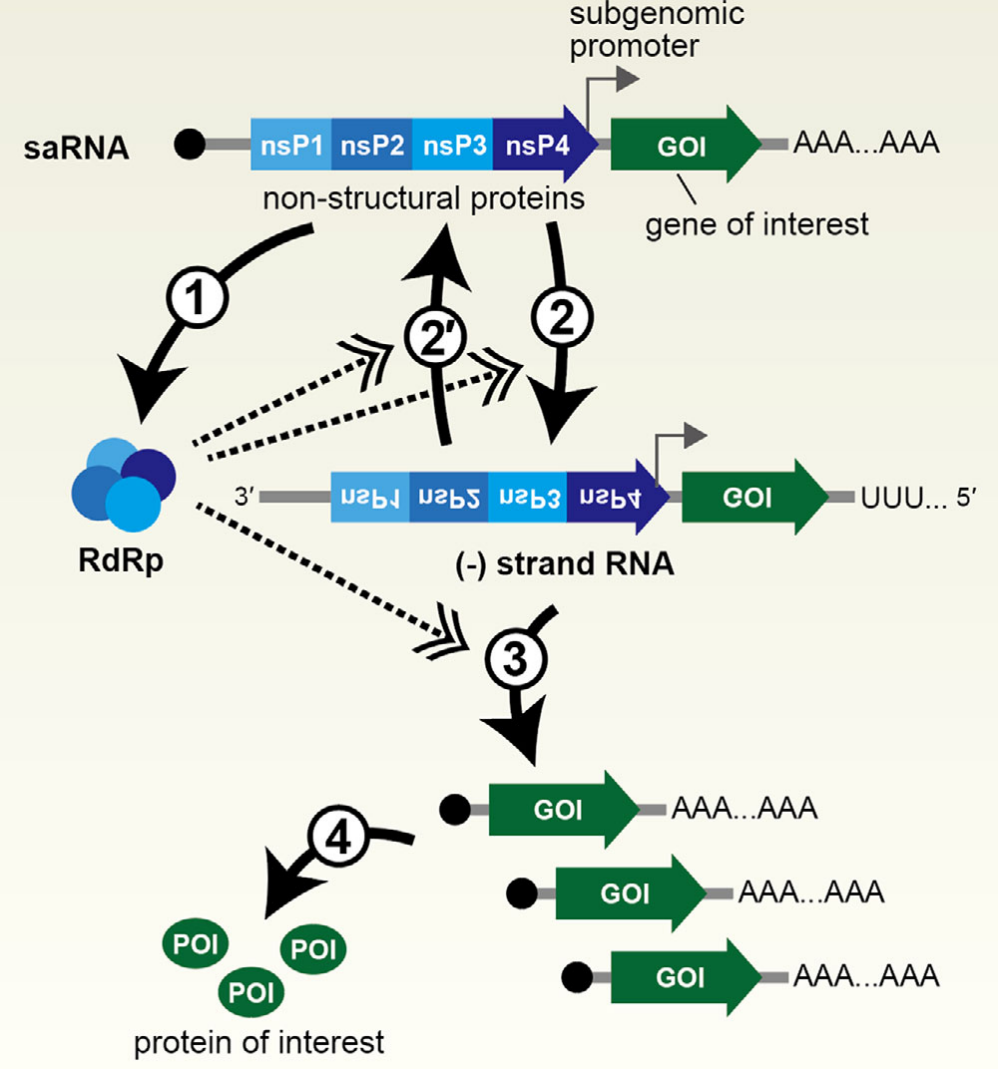
Self‐amplifying RNA (saRNA). The saRNA encodes nonstructural proteins (nsPs) derived from alphaviruses together with a gene of interest (GOI). Nonstructural proteins, which possess RNA‐dependent RNA polymerase (RdRp) activity, are expressed (1). Using the saRNA as a template, the RdRp synthesizes a complementary (‐) strand RNA (2), which in turn serves as a template for generating new (+) strand saRNA (2′). In addition, the RdRp synthesizes a subgenomic RNA from the region downstream of the subgenomic promoter (3), enabling expression of the protein of interest (4).

When used as gene delivery vectors, self‐amplifying RNA is typically based on systems derived from alphaviruses, which possess a positive‐sense single‐stranded RNA genome. Venezuelan Equine Encephalitis Virus (VEEV), Semliki Forest Virus (SFV), and Sindbis Virus (SINV) are popular alphaviruses to create such systems [66, 67]. When self‐amplifying RNA is introduced into a cell, non‐structural proteins (nsPs), which mediate RNA amplification, are generated. These NSPs exhibit RNA‐dependent RNA polymerase activity, allowing the synthesis of negative (‐, minus) strand RNA from the positive‐sense (+, plus) strand template, and subsequently the production of new (+) strand RNA using the negative strand as a template. Furthermore, subgenomic RNA is synthesized from the subgenomic promoter of (‐) strand RNA. By inserting an exogenous gene into this subgenomic region, sustained expression of the transgene can be achieved (Figure 4).

Vaccine applications for various cancers and infectious diseases caused by the Ebola virus, HIV, Zika virus, and malaria are in progress [68]. In November 2023, a saRNA vaccine for COVID‐19 became the first of its kind to be approved [69]. The use of self‐amplifying RNA not only offers greater persistence but also minimizes adverse reactions by reducing its dose. Due to its high safety and persistence, saRNA has also been explored as a vector for expressing Yamanaka factors to reprogram human somatic cells into iPS cells. In conventional mRNA‐based approaches, multiple rounds of transfection are needed due to the low persistence of mRNA. In contrast, saRNA enables iPS cell generation with a single transfection [70, 71].

Self‐amplifying RNA typically loses its function when conventional base modifications such as pseudouridine (ψ) or *N* ^1^‐methylpseudouridine (m^1^ψ), which are commonly used in synthetic mRNAs, are introduced. Consequently, reducing immunogenicity through base modification is not feasible, and in some cases, cytotoxicity has been a concern. However, recent studies have identified base modifications tolerated in saRNA [72, 73]. Substitution of cytidine (C) with 5‐methylcytidine (m^5^C) does not inhibit self‐amplification or transgene expression but effectively suppresses interferon responses upon transfection. Similar to the role of m^1^ψ in synthetic mRNA, m^5^C may become a standard modification in self‐amplifying RNA.

Since the NSP coding region alone accounts for approximately 7.5 kilobases (kb), self‐amplifying RNAs are larger in size. The total length often exceeds 10 kb, and it may approach 20 kb, depending on the size of the inserted gene [71]. This substantial length poses challenges in synthesis, purification, and quality management. To address this problem, a divided system has been developed in which the NSP‐coding mRNA and the trans‐amplifying RNA, replicated by the NSPs, are expressed separately [74, 75, 76]. This trans‐amplifying RNA (taRNA) strategy offers several practical advantages, including reduced RNA component size, improved manufacturability, and greater flexibility in optimizing the replicase and the transgene separately. In addition, separating the replicase from the gene of interest can improve safety by limiting the persistence of replication activity and enabling tighter control over amplification [77]. At present, however, classical self‐amplifying RNAs remain more advanced in clinical development, with multiple vaccine candidates having entered or completed clinical trials [78]. Nevertheless, as taRNA platforms continue to mature, their modularity and safety features position them as a promising next‐generation alternative for future translational applications.

Additional strategies have also been explored, including directed evolution of NSP variants [79], to leverage naturally occurring mutations in RNA transfected into cells. In this approach, RNA is transfected into IFN‐responsive cells that mimic host immunity and maintained in long‐term culture. Among the resulting mutant RNAs, NSP mutants that have enhanced expression from the subgenomic region are selected and subsequently transfected into new cells. By repeating this selection cycle, variants with improved expression levels and prolonged stability could be obtained. Beyond such sequence optimization of viral replicase proteins, an alternative strategy is to introduce temperature‐sensitive mutations into NSPs [80]. An NSP mutant isolated from a systematic mutagenesis and screening campaign exhibited temperature‐dependent inactivation at 37°C, suggesting a safety switch that restricts replicon activity to lower‐temperature tissues such as the skin, and thereby providing a potential platform for safer vaccine applications. In addition to alphavirus‐derived replicons, alternative self‐amplifying RNA backbones, including flavivirus‐based systems [81], have also been explored as potential platforms for transgene expression.

Despite such advances, self‐amplifying RNA still presents important trade‐offs compared with conventional modified mRNA. saRNA generally exhibits higher reactogenicity, greater manufacturing complexity, and more stringent quality control requirements due to its large size and replication competence. Moreover, controlling the duration and magnitude of expression remains challenging, and the generation of replication‐competent byproducts during manufacturing requires careful monitoring. Lastly, because saRNA encodes viral nonstructural proteins, it may elicit T‐cell responses against NSPs, which could further influence safety and tolerability profiles. Given its potential to overcome the instability and limited duration of conventional mRNA, self‐amplifying RNA is considered one of the most promising modalities for the future.

### 3.4 Lantern‐Shaped RNA

**FIGURE 5:**
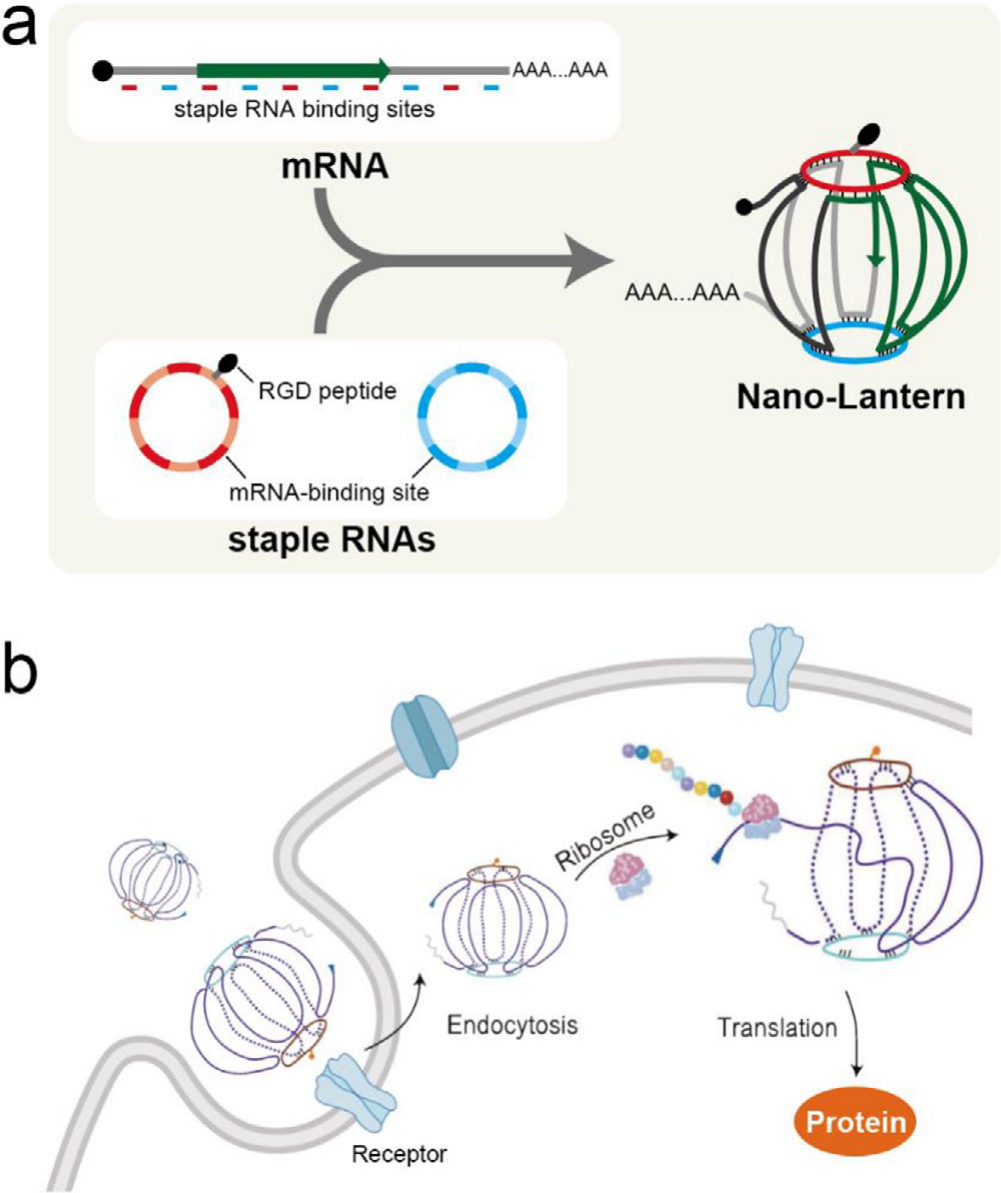
Schematic illustration of the “Nano‐Lantern” mRNA system. (a) Formation of a compact spherical mRNA nanoparticle by hybridization of an mRNA with two staple RNAs carrying complementary sequences and an RGD peptide. (b) Cellular uptake and translation of the Nano‐Lantern mRNA. Panel (b) is adapted from Hu et al. [83], licensed under CC BY 4.0.

In the field of bionanotechnology, RNA has been used as a construction material to assemble diverse nanoscale architectures of different shapes and sizes [82]. Here, we highlight an example that folds an artificial mRNA itself into a compact, spherical particle. Hu et al. hybridized a therapeutic mRNA with two staple RNAs carrying sequences partially complementary to the mRNA, thereby folding it into a compact sphere termed a nano‐lantern (Figure 5) [83]. This lantern‐like framework increased the stability of artificial mRNA in serum while preserving translational competence. The staple RNAs were further appended with an RGD peptide to target receptors overexpressed on colorectal cancer cells. The nano‐lantern, with a compact design and RGD peptide, was efficiently taken up by colorectal cancer cells in mice, thereby enabling expression of mRNA‐encoded SMAD4 and yielding therapeutic benefits.

Currently, synthetic mRNAs are commonly delivered with lipid nanoparticles (LNPs) to facilitate cellular entry. In this study, however, the nano‐lantern was administered as naked RNA, suggesting that particular structures permit uptake of RNA alone. Notably, naked mRNA has been reported to achieve relatively efficient delivery to cardiac cells following direct local administration [84, 85]. However, in general, naked mRNA is considered difficult to deliver in vivo because of rapid degradation, poor cellular uptake, and innate immune sensing, and such successes appear to be highly model‐ and route‐dependent [86]. Whether structural compaction into a nano‐lantern can overcome these intrinsic barriers in other tissues or via systemic administration remains an open and important question.

Additional functions could be encoded on staple RNAs. For example, embedding RNA aptamers that bind specific proteins could direct subcellular localization. Alternatively, staples designed to hybridize with specific endogenous RNAs could relax the therapeutic mRNA and promote translation, whereas promoting strong secondary structure formation could reduce translation. Further advances are possible as these strategies are refined and validated across relevant models.

### 3.5 Poly(A)‐Downstream Engineering

**FIGURE 6:**
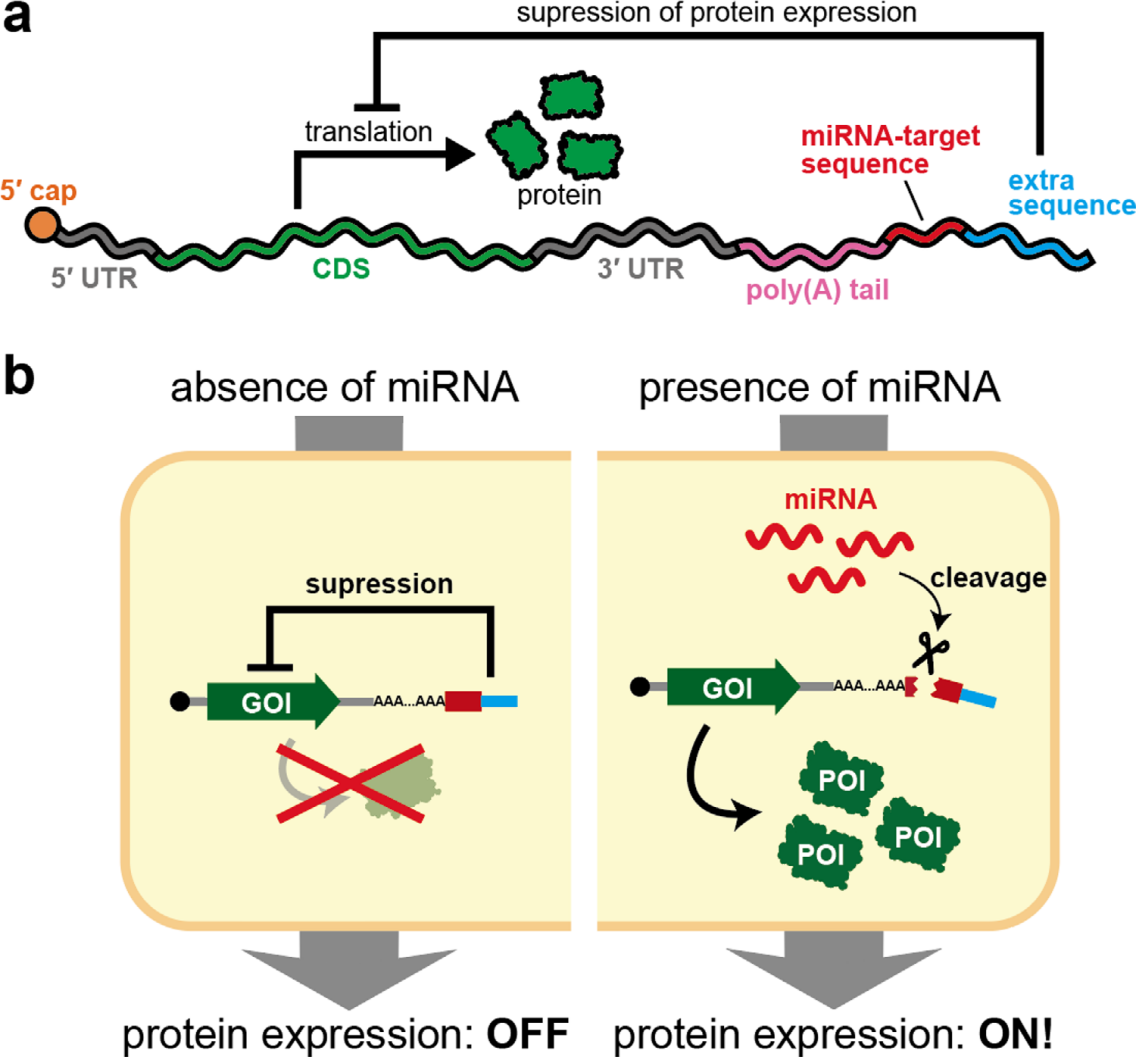
microRNA‐responsive ON switch. (a) A synthetic mRNA carries a miRNA target sequence downstream of the poly(A) tail and an extra inhibitory sequence. In cells lacking the miRNA, protein expression is suppressed; whereas in cells expressing the target miRNA, cleavage of the target site activates protein expression. (b) In the absence (left) or presence (right) of the miRNA, expression of the protein of interest (POI) is, respectively, turned OFF or ON.

Finally, we introduce an RNA structure‐based system for controlling translation developed by our group. Fujita et al. found that appending a non‐poly(A) extra sequence downstream of the poly(A) tail suppresses protein expression and designed a microRNA‐responsive ON switch utilizing this mechanism [87]. In such a construct, a fully complementary site for an arbitrary miRNA is placed immediately downstream of the poly(A) tail, followed by an inhibitory sequence that suppresses protein expression (Figure 6a). In the absence of the target miRNA, the appended 3′ sequence keeps protein expression OFF; when the target miRNA is present, cleavage at the complementary site on the synthetic mRNA removes the appended sequence and, as a result, turns protein expression ON (Figure 6b). Consequently, exogenous genes can be expressed specifically in cells with certain miRNAs. This system enables the selection of target cells based on their miRNA profiles if the exogenous gene encodes an apoptosis‐inducing protein. However, there is a limitation of this miRNA‐responsive ON switch in that repression by the appended sequence may be inadequate, thereby leading to expression leakages even in the OFF state. To address this problem, Fujita et al. co‐expressed an OFF switch encoding a target‐inhibiting protein. Nevertheless, this strategy is only applicable when the target protein has a suitable inhibitor.

To achieve a versatile and robust gene‐expression control system, Masaki et al. developed a hybrid switch that embeds both OFF and ON switch elements within a single synthetic mRNA [88]. As an alternative, a split switch incorporating a post‐translational control module has also been designed to improve the ON/OFF ratio (Figure 6c) [89]. Moreover, ongoing studies aim to elucidate the mechanism by which appended 3′ sequences suppress translation and further optimize the ON switch to refine the artificial RNA switch system.

By enabling gene expression to respond to endogenous cellular states, such as microRNA profiles, these switches provide a safety and specificity layer that cannot be achieved by delivery control alone. Importantly, they are complementary to advances in targeted RNA delivery, such that physical targeting (e.g., ligand‐ or tissue‐directed delivery systems) can be combined with post‐delivery, cell‐intrinsic translational control to achieve multi‐layered specificity. This dual‐control strategy is particularly valuable for therapeutic applications requiring precise, cell‐selective expression, and highlights how synthetic mRNA can evolve from a simple expression vector into a programmable and context‐responsive molecular system.

## 4 Beyond the Art of Nature: Outlook for Artificial mRNA

Proteins orchestrate virtually all biological phenomena, and messenger RNA—the molecule encoding them—holds the potential to influence every aspect of life. Indeed, synthetic mRNA applications are rapidly expanding, encompassing infectious disease vaccines, cancer immunotherapy, gene and cell therapy, genome editing, and regenerative medicine such as CAR‐T cell engineering. This remarkable progress has been driven by cumulative advances across diverse disciplines, including classical mRNA biology, immunology, virology, nucleic acid chemistry, organic synthesis, and synthetic biology. Equally essential are innovations in RNA synthesis, purification, and delivery technologies, which have successfully transitioned from concept to clinical reality. As synthetic mRNA continues to evolve beyond its natural design—becoming circular, branched, self‐amplifying, or even programmable in its translation control—we may soon witness entirely new forms of molecular expression.

At the same time, these emerging architectures introduce new practical challenges that must be addressed before broad translational adoption. Compared with conventional linear mRNA, non‐canonical formats—such as circular, branched, self‐amplifying, and structurally folded RNAs—often require more complex synthesis workflows, specialized enzymatic or chemical ligation steps, and more stringent purification processes. Structural heterogeneity, incomplete processing, residual reaction components, and batch‐to‐batch variability can complicate quality control and regulatory comparability. Moreover, differences in capping efficiency, poly(A) tail length distributions, dsRNA contamination, transcript integrity, and incomplete incorporation of modified nucleotides can profoundly affect translational output, immunogenicity, and biological interpretation. As chemically and structurally complex mRNAs become more widely adopted, rigorous analytical validation—such as LC–MS, cap analysis, electrophoretic profiling, and dsRNA quantification—will be essential for ensuring reproducibility and safety. Thus, while non‐canonical mRNAs offer powerful new capabilities, their practical implementation will require parallel advances in scalable manufacturing, quality control, and regulatory frameworks.

The future of artificial mRNA will be shaped by the creative efforts of researchers from many fields, and its potential is limited only by our imagination.

## Conflicts of Interest

H.S. is an inventor on patents related to microRNA‐responsive RNA switches, and is a co‐founder of aceRNA Technologies, a company pursuing applications of these technologies. Other authors declare no conflict of interest.
